# Supplementary material for: Deep IDA: A Deep Learning Method for Integrative Discriminant Analysis of Multi-View Data with Feature Ranking–An Application to COVID-19 severity
Source: ArXiv. 2021 Nov 18:arXiv:2111.09964v1. Preprint. [Version 1] (PMC8609900)
Supplement: Supplementary file 1 [file NIHPP2111.09964V1-supplement-1.pdf]

arXiv:2111.09964v1 [stat.ML] 18 Nov 2021

Supplementary Material for "Deep IDA: A Deep Learning Method for Integrative Discriminant Analysis of Multi-View Data with Feature Ranking–An Applicaition to COVID-19 severity"

Jiuzhou Wang, Sandra E. Safo
Division of Biostatistics
University of Minnesota, MN

1 Theorems and Proofs

Theorem 1. *Let $\mathbf{S}_t^d$ and $\mathbf{S}_b^d$ respectively be the total covariance and the between-class covariance for the top-level representations $\mathbf{H}^d, d = 1, \ldots, D$. Let $\mathbf{S}_{dj}$ be the cross-covariance between top-level representations d and j. Assume $\mathbf{S}_t^d \succ 0$. Define $\mathcal{M}^d = \mathbf{S}_t^{d^{-\frac{1}{2}}}\mathbf{S}_b^d\mathbf{S}_t^{d^{-\frac{1}{2}}}$ and $\mathcal{N}_{dj} = \mathbf{S}_t^{d^{-\frac{1}{2}}}\mathbf{S}_{dj}\mathbf{S}_t^{j^{-\frac{1}{2}}}$. Then $\mathbf{\Gamma}^d \in \Re^{o_d \times l}$, $l \leq \min\{K-1, o_1, \ldots, o_D\}$ in equation (4) of main text are eigenvectors corresponding respectively to eigenvalues $\mathbf{\Lambda}_d = diag(\lambda_{d_k}, \ldots, \lambda_{d_l})$, $\lambda_{d_k} > \cdots > \lambda_{d_l} > 0$ that iteratively solve the eigensystem problems:*

$$\left(c_1\mathcal{M}^d + c_2\sum_{j\neq d}^{D}\mathcal{N}_{dj}\mathbf{\Gamma}_j\mathbf{\Gamma}_j^T\mathcal{N}_{dj}^T\right)\mathbf{\Gamma}_d = \mathbf{\Lambda}_d\mathbf{\Gamma}_d, \forall d = 1, ..., D$$

where $c_1 = \frac{\rho}{D}$ and $c_2 = \frac{2(1-\rho)}{D(D-1)}$.

Prove. Solving the optimization problem is equivalent to iteratively solving the following generalized eigenvalue systems:

$$\left(c_1\mathcal{M}^1 + c_2\sum_{j=2}^{D}\mathcal{N}_{1j}\mathbf{\Gamma}_j\mathbf{\Gamma}_j^T\mathcal{N}_{1j}^T\right)\mathbf{\Gamma}_1 = \mathbf{\Lambda}_1\mathbf{\Gamma}_1$$

$$\vdots$$

$$\left(c_1\mathcal{M}^d + c_2\sum_{j=1, j\neq d}^{D}\mathcal{N}_{dj}\mathbf{\Gamma}_j\mathbf{\Gamma}_j^T\mathcal{N}_{dj}^T\right)\mathbf{\Gamma}_d = \mathbf{\Lambda}_d\mathbf{\Gamma}_d$$

$$\vdots$$

$$\left(c_1\mathcal{M}^D + c_2\sum_{j=1}^{D-1}\mathcal{N}_{Dj}\mathbf{\Gamma}_j\mathbf{\Gamma}_j^T\mathcal{N}_{Dj}^T\right)\mathbf{\Gamma}_D = \mathbf{\Lambda}_D\mathbf{\Gamma}_D$$

where $c1 = \frac{\rho}{D}$ and $c2 = \frac{2(1-\rho)}{D(D-1)}$.

Proof. The Lagrangian is

$$\begin{aligned} L(\mathbf{\Gamma}_1, ..., \mathbf{\Gamma}_D, \lambda_1, ..., \lambda_D) &= \rho\frac{1}{D}\sum_{d=1}^{D} tr[\mathbf{\Gamma}_d^T\mathcal{M}^d\mathbf{\Gamma}_d] + (1-\rho)\frac{2}{D(D-1)}\sum_{d=1}^{D}\sum_{j,j\neq d}^{D} tr[\mathbf{\Gamma}_d^T\mathcal{N}_{dj}\mathbf{\Gamma}_j\mathbf{\Gamma}_j^T\mathcal{N}_{dj}^T\mathbf{\Gamma}_d] - \sum_{d=1}^{D}\eta_d(tr[\mathbf{\Gamma}_d^T\mathbf{\Gamma}_d] - l) \\ &= c_1\sum_{d=1}^{D} tr[\mathbf{\Gamma}_d^T\mathcal{M}^d\mathbf{\Gamma}_d] + c_2\sum_{d=1}^{D}\sum_{j,j\neq d}^{D} tr[\mathbf{\Gamma}_d^T\mathcal{N}_{dj}\mathbf{\Gamma}_j\mathbf{\Gamma}_j^T\mathcal{N}_{dj}^T\mathbf{\Gamma}_d] - \sum_{d=1}^{D}\lambda_d(tr[\mathbf{\Gamma}_d^T\mathbf{\Gamma}_d] - l) \end{aligned} \quad (1)$$

The first order stationary solution for $\mathbf{\Gamma}_d (\forall d = 1, ..., D)$ is

$$\frac{\partial L(\mathbf{\Gamma}_1, ..., \mathbf{\Gamma}_D, \lambda_1, ..., \lambda_D)}{\partial \mathbf{\Gamma}_d^T} = 2c_1\mathcal{M}^d\mathbf{\Gamma}_d + 2c_2\sum_{j,j\neq d}^{D}(\mathcal{N}_{dj}\mathbf{\Gamma}_j\mathbf{\Gamma}_j^T\mathcal{N}_{dj}^T)\mathbf{\Gamma}_d - 2\lambda_d\mathbf{\Gamma}_d = \mathbf{0} \quad (2)$$

Rearranging the equation 2 we have

$$\left(c_1\mathcal{M}^d + c_2\sum_{j,j\neq d}^{D}\mathcal{N}_{dj}\mathbf{\Gamma}_j\mathbf{\Gamma}_j^T\mathcal{N}_{dj}^T\right)\mathbf{\Gamma}_d = \lambda_d\mathbf{\Gamma}_d$$

For $\mathbf{\Gamma}_j, \forall j \neq d$ fixed, the above can be solved for the eigenvalues of $(c_1\mathcal{M}^d + c_2\sum_{j,j\neq d}^{D}\mathcal{N}_{dj}\mathbf{\Gamma}_j\mathbf{\Gamma}_j^T\mathcal{N}_{dj}^T)$. Arrange the eigenvalues from large to small and denote $\mathbf{\Lambda}_d \in \mathbf{R}^{o_d\times o_d}$ as the diagonal matrix of those values. For the top l largest eigenvalues, denote the corresponding eigenvectors as $\widehat{\mathbf{\Gamma}}_d = [\gamma_{d,1}, ..., \gamma_{d,l}]$. Therefore, starting from $d = 1$, following this process, $\widehat{\mathbf{\Gamma}}_1$ is updated; then, update $\widehat{\mathbf{\Gamma}}_2$ and so on; finally, update $\widehat{\mathbf{\Gamma}}_D$. We iterate until convergence, which is defined as, $\frac{\|\widehat{\mathbf{\Gamma}}_{d,new} - \widehat{\mathbf{\Gamma}}_{d,old}\|_F^2}{\|\widehat{\mathbf{\Gamma}}_{d,old}\|_F^2} < \epsilon$. When convergence is achieved, set $\widetilde{\mathbf{\Gamma}}_d = \widehat{\mathbf{\Gamma}}_d, \forall d = 1, ..., D$. ∎

Theorem 2. *For d fixed, let $\eta_{d,1}, \ldots, \eta_{d,l}$, $l \leq \min\{K-1, o_1, \ldots, o_D\}$ be the largest l eigenvalues of $c_1\mathcal{M}^d + c_2\sum_{j\neq d}^{D}\mathcal{N}_{dj}\mathbf{\Gamma}_j\mathbf{\Gamma}_j^T\mathcal{N}_{dj}^T$. Then the solution $\widetilde{f}^d$ to the optimization problem in equation (5) [main text] for view d maximizes*

$$\sum_{r=1}^{l}\eta_{d,r}. \tag{3}$$

Proof. Fix d and let $\eta_{d,1}, \eta_{d,2}, ..., \eta_{d,l}$ be the top l eigenvalues of

$$c_1\mathcal{M}^d + c_2\sum_{j\neq d}^{D}\mathcal{N}_{dj}\widetilde{\mathbf{\Gamma}}_j\widetilde{\mathbf{\Gamma}}_j^T\mathcal{N}_{dj}^T.$$

Then,

$$\sum_{r=1}^{l}\eta_{d,r} = c_1 tr[\widetilde{\mathbf{\Gamma}}_d^T\mathcal{M}^d\widetilde{\mathbf{\Gamma}}_d] + c_2\sum_{j,j\neq d}^{D} tr[\widetilde{\mathbf{\Gamma}}_d^T\mathcal{N}_{dj}\widetilde{\mathbf{\Gamma}}_j\widetilde{\mathbf{\Gamma}}_j^T\mathcal{N}_{dj}^T\widetilde{\mathbf{\Gamma}}_d]$$

Proof.

$$\begin{aligned}
&c_1 tr[\widetilde{\mathbf{\Gamma}}_d^T\mathcal{M}^d\widetilde{\mathbf{\Gamma}}_d] + c_2\sum_{j,j\neq d}^{D} tr[\widetilde{\mathbf{\Gamma}}_d^T\mathcal{N}_{dj}\widetilde{\mathbf{\Gamma}}_j\widetilde{\mathbf{\Gamma}}_j^T\mathcal{N}_{dj}^T\widetilde{\mathbf{\Gamma}}_d]\\
&= tr(\widetilde{\mathbf{\Gamma}}_d^T(c_1\mathcal{M}^d + c_2\sum_{j,j\neq d}^{D}\mathcal{N}_{dj}\widetilde{\mathbf{\Gamma}}_j\widetilde{\mathbf{\Gamma}}_j^T\mathcal{N}_{dj}^T)\widetilde{\mathbf{\Gamma}}_d)\\
&= tr(\widetilde{\mathbf{\Gamma}}_d^T\mathbf{\Lambda}_d\widetilde{\mathbf{\Gamma}}_d)\\
&= \sum_{r=1}^{l}\eta_{d,r}
\end{aligned}$$

∎

2 More Results From Real Data Analysis

2.1 Data preprocessing and application of Deep IDA and competing methods

Of the 128 patients, 125 had both omics and clinical data. We focused on proteomics, RNA-seq, and metabolomics data in our analyses since many lipids were not annotated. We formed a four-class classification problem using COVID-19 and ICU status. Our four groups were: with COVID-19 and not admitted to the ICU (COVID Non-ICU), with COVID-19 and admitted to the ICU (COVID ICU), no COVID-19 and admited to the ICU (Non-COVID ICU), and no COVID-19 and not admitted to the ICU (Non-COVID Non-ICU). The frequency distribution of samples in these four groups were: 40% COVID ICU, 40% COVID Non-ICU, 8% Non-COVID Non-ICU, and 12% Non-COVID ICU. The initial dataset contains 18,212 genes, 517 proteins, and 111 metabolomics features. Prior to applying our method, we pre-processed the data as follows. All genes which were missing in our samples were removed from the dataset and 15,106 genes remained. We selected genes that more than half of their variables are non-zero, and we applied box-cox transformation on each gene as the gene data were highly

skewed. The transformed data were standardized to have mean zero and variance one. We kept genes with variance less than the 25th percentile. We then used ANOVA on the standardized data to filter out (p-values > 0.05) genes with low potential to discriminate among the four groups. For the proteomics and metabolomics data, we standardized each molecule to have mean zero and variance one, pre-screened with ANOVA and filtered out molecules with p-values > 0.05. Our final data were $\mathbf{X}^1 \in \Re^{125\times 2,734}$ for the gene data, $\mathbf{X}^2 \in \Re^{125\times 269}$ for the protoemics data, and $\mathbf{X}^3 \in \Re^{125\times 66}$ for the metabolomics data.

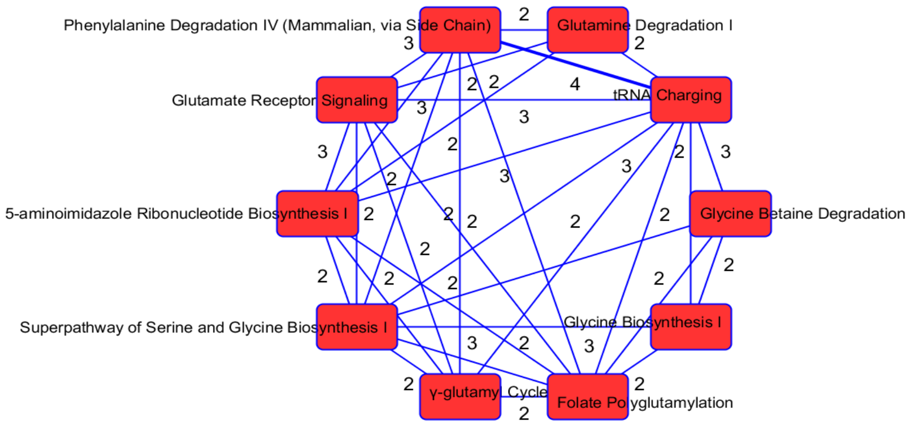

Figure 1: Network of overlapping canonical pathways from highly ranked metabolites. Nodes refer to pathways and a line connects any two pathways when there is at least two molecules in common between them.

Table 1: Top Diseases and Biological Functions from Ingenuity Pathway Analysis (IPA).

	Top Diseases and Bio Functions	P-value range	Molecules Selected
RNA Sequencing	Cancer (such as non-melanoma solid tumor, head and neck tumor)	4.96E-02 – 2.74E-05	48
	Organismal Injury and Abnormalities	4.96E-02 – 2.74E-05	48
	Neurological Disease (such as glioma cancer, brain lesion, neurological deficiency)	4.86E-02 – 5.84E-05	36
	Developmental Disorder (such as intellectual diability with ataxia)	4.46E-02 – 3.76E-05	16
	Hereditary Disorder (such as familial midline effect)	4.86E-02 – 2.02E-05	16
Proteomics	Infectious Diseases (such as Severe COVID-19, COVID-19, infection by SARS coronavirus)	1.75E-03 – 8.31E-13	19
	Inflammatory Response (such as inflammation of organ, degranulation of blood platelets)	1.29E-03 – 1.34E-12	32
	Metabolic Disease (such as amyloidosis, Alzheimer disease, diabetes mellitus)	1.47E-03 – 2.48E-12	20
	Organismal Injury and Abnormalities (such as amyloidosis, tauopathy)	1.72E-03 – 2.48E-12	39
	Neurological Disease (such as tauopathy, progressive encephalopathy, progressive neurological disorder)	1.57E-03 – 3.41E-11	34
Metabolomics	Cancer	3.63E-02 – 5.20E-14	18
	Gastrointestinal Disease (such as digestive system cancer, hepatocellular carcinoma)	3.64E-02 – 5.20E-14	20
	Organismal Injury and Abnormalities (such as digestive system cancer, abdominal cancer)	3.79E-02 – 5.20E-14	22
	Hepatic System Disease (such as hepatocellular carcinoma, liver lesion)	2.91E-02 – 1.66E-11	15
	Developmental Disorder (such as mucopolysaccharidosis type I, spina bifida)	2.44E-02 – 1.83E-09	11

Table 2: Top Molecular and Cellular Functions Functions from Ingenuity Pathway Analysis (IPA).

	Molecular and Cellular Functions	P-value range	Molecules Selected
RNA Sequencing	Cell Death and Survival	4.46E-02 – 2.00E-03	8
	Amino Acid Metabolism	3.47E-02 – 2.07E-05	2
	Cell-to-cell Signaling and Interaction	4.86E-02 – 2.07E-03	10
	Cellular Assembly and Organization	4.46E-02 – 2.07E-03	9
	Cellular Function and Maintenance	4.86E-02 – 2.07E-03	10
Proteomics	Cellular Compromise	1.29E-03 – 1.34E-12	13
	Cellular Movement	1.65E-03 – 2.19E-09	24
	Lipid Metabolism	1.28E-03 – 2.95E-09	15
	Molecular Transport	1.28E-03 – 2.95E-09	15
	Small molecule Biochemistry	1.61E-03 – 2.95E-09	19
Metabolomics	Amino Acid Metabolism	3.64E-02 – 3.99E-08	9
	Molecular Transport	3.82E-02 – 3.99E-08	17
	Small Molecule Biochemistry	3.64E-02 – 3.99E-08	18
	Cellular Growth and Proliferation	3.79E-02 – 5.12E-08	16
	Cell Cycle	3.63E-02 – 5.81E-07	10

Table 3: **Linear Simulations** Network structures for all deep learning based methods. In order to make fair comparisons, for each dataset, the network structure for Deep CCA/Deep GCCA is the same as the proposed Deep IDA method. The activation function is Leakly Relu with parameter 0.1 by default. After activation, batch normalization is also implemented. $-$ indicates not applicable

Data	Sample size (Train,Valid,Test)	Feature size (p^1, p^2, p^3)	Method	Network structure	Epochs per run	Batch size
Setting 1	540,540,1080	1000,1000,-	Deep IDA (+Bootstrap)	Input-512-256-64-10	50	540
Setting 1	540,540,1080	1000,1000,-	Deep CCA	Input-512-256-64-10	50	180
Setting 2	540,540,1080	1000,1000,1000	Deep IDA (+Bootstrap)	Input-512-256-20	50	540
Setting 2	540,540,1080	1000,1000,1000	Deep GCCA	Input-512-256-64-20	200	540

Table 4: **Nonlinear Simulations** Network structures for all deep learning based methods. In order to make fair comparisons, for each dataset, the network structure for Deep CCA/Deep GCCA is the same as the proposed Deep IDA method. The activation function is Leakly Relu with parameter 0.1 by default. After activation, batch normalization is also implemented.

Data	Sample size (Train,Valid,Test)	Feature size (p^1, p^2, p^3)	Method	Network structure	Epochs per run	Batch size
Setting 1	350,350,350	500,500	Deep IDA (+Bootstrap)	Input-256*10-64-20	50	350
Setting 1	350,350,350	500,500	Deep CCA	Input-256*10-64-20	50	350
Setting 3b	5250,5250,5250	500,500	Deep IDA (+Bootstrap)	Input-256-256-256-256-256-256-64-20	50	500
Setting 3b	5250,5250,5250	500,500	Deep CCA	Input-256-256-256-256-256-256-64-20	50	500
Setting 4	350,350,350	2000,2000	Deep IDA (+Bootstrap)	input-256-256-256-256-256-256-256-64-20	50	350
Setting 4	350,350,350	2000,2000	Deep CCA	input-256-256-256-256-256-256-256-64-20	50	350
Setting 5b	5250,5250,5250	2000,2000	Deep IDA (+Bootstrap)	Input-256-256-256-256-256-256-64-20	50	500
Setting 5b	5250,5250,5250	2000,2000	Deep CCA	Input-256-256-256-256-256-256-64-20	50	500

Table 5: **Real Data Analysis** Network structures for all deep learning based methods. In order to make fair comparisons, for each dataset, the network structure for Deep CCA/Deep GCCA is the same as the proposed Deep IDA method. The activation function is Leakly Relu with parameter 0.1 by default. After activation, batch normalization is also implemented. For Covid-19 data, we select the top 50 features for each view from Bootstrap Deep IDA with input-512-20. $-$ indicates not applicable

Data	Sample size (Train,Valid,Test)	Feature size (p^1, p^2, p^3)	Method	Network structure	Epochs per run	Batch size
Noisy MNIST	50000,10000, 10000	784,784,-	Deep CCA non-Bootstrap Deep IDA	Input-512-256-64-20	50	50000
Covid-19	74,0,21	2734,269,66	non-Bootstrap Deep IDA	Input-512-20	20	74
Covid-19	74,0,21	2734,269,66	Deep IDA on selected top 50 features	Input-512-256-20	20	74
Covid-19	74,0,21	2734,269,66	Deep IDA on selected top 10 percent features	Input-256-64-20	20	74
Covid-19	74,0,21	2734,269,66	Deep GCCA on selected top 50 features	Input-256-20	150	74
